# Determining the Shear Capacity of Steel Beams with Corrugated Webs by Using Optimised Regression Learner Techniques

**DOI:** 10.3390/ma14092364

**Published:** 2021-05-01

**Authors:** Ahmed S. Elamary, Ibrahim B. M. Taha

**Affiliations:** 1 Civil Engineering Department, College of Engineering, Taif University, P.O. Box 11099, Taif 21944, Saudi Arabia; 2 Electrical Engineering Department, College of Engineering, Taif University, P.O. Box 11099, Taif 21944, Saudi Arabia; i.taha@tu.edu.sa

**Keywords:** shear strength, corrugated web, regression learner techniques, steel beams

## Abstract

The use of corrugated webs increases web shear stability and eliminates the need for transverse stiffeners in steel beams. Optimised regression learner techniques (ORLTs) are rarely used for calculating shear capacity in steel beam research. This study proposes a new approach for calculating the maximum shear capacity of steel beams with trapezoidal corrugated webs (SBCWs) by using ORLTs. A new shear model is proposed using ORLTs in accordance with plate buckling theory and previously developed formulas for predicting the shear strength of SBCWs. The proposed ORLT models are implemented using the regression learner toolbox of MATLAB software (2020b). The available data of more than 125 test results from different specimens prepared by previous researchers are used to create the model. In this study, web geometry and relevant web steel grades determine the shear capacity of SBCWs. Four regression methods are adopted. Results are compared with those of an artificial neural network model. The model output factor represents the ratio of the web vertical shear stress to the normalised shear stress. Shear capacity can be estimated on the basis of the resulting factor from the model. The proposed model is verified using two methods. In the first method, a series of tests are performed by the authors. In the second method, the results of the model are compared with the shear values obtained experimentally by other researchers. On the basis of the test results of previous studies and the current work, the proposed model provides an acceptable degree of accuracy for predicting the shear capacity of SBCWs. The results obtained using Gaussian process regression are the most appropriate because its recoded mean square error is 0.07%. The proposed model can predict the shear capacity of SBCWs with an acceptable percentage of error. The recoded percentage of error is less than 5% for 93% of the total specimens. By contrast, the maximum differential obtained is ±10%, which is recorded for 3 out of 125 specimens.

## 1. Introduction

Extensive studies have been conducted regarding the shear strength (SS) of steel beams with trapezoidal corrugated webs (SBCWs). This section presents some of these studies. An experimental study was conducted by Lindner and Aschinger [1] to calculate the SS of SBCWs. They suggested using 70% of shear buckling stress as the nominal SS for designing SBCWs. Worthy experimental and analytical research conducted by Elgaaly et al. [2], using loaded predominantly in shear. Large-scale SBCW investigations were conducted by Sause et al. [3], Abbas [4] and Driver et al. [5] to estimate the SS of SBCWs. They provided an equation for estimating the lower bound of the SS of SBCWs. In addition, they recommended precluding global buckling because this phenomenon requires a significant loss of strength and a low degree of post-buckling strength. Yi et al. [6] presented a formula for the nominal SS of SBCWs. This formula was validated by comparing the obtained values of 15 test results and finite element analysis results. Moon et al. [7] reported the results of three tests and described the SS formula presented by Yi et al. [6]. Moon et al. [7] compared the results of their proposed formula with the results of several formulas developed by other researchers from 17 tests. Sause and Braxtan [8] theoretically investigated the SS of SBCWs. They collected a database of 102 tests from 8 previous studies and developed an analytical model for estimating the normalised SS. Their formula was consistent with only one subset (i.e., 22 test results) of the 120 available published test results. These researchers attributed the inconsistency to the test conditions. Consequently, their proposed model was valid only for SBCWs that fulfil the geometric criteria they set. From the previous research, numerous buckling formulas have been proposed to calculate global shear buckling and interaction buckling (IB). The proposed formulas for calculating IB were given by [6,8,9,10,11,12,13] from 1984 to 2008. Regarding the hybrid steel beams with corrugated web, Elamary et al. [14] presented an experimental study concerned with the failure mechanism of SBCW’s non-welded inclined fold. The case of non-welded inclined folds, owing to decrease the effect of the fatigue cracks initiated along the inclined folds. Additionally, they studied the influence of using a limited number of flange stiffeners at certain places to postpone the earlier flange buckling that may occur in these places.

Extensive research was conducted and focus on the computational methods and their uses for validating experiments; some of it is presented herein, which carried out by [15,16,17,18,19,20]. Manoj et al. [15] studied the flexural behaviour of steel beams by using ANSYS software. They reported that the load-carrying capacity of the CW beam increased by increasing the web thickness as well as the optimum corrugated angle is recommended to be 45°. Krejsa et al. [16] and Čajka et al. [17,18] discussed an application of the original and probabilistic method—“Direct Optimized Probabilistic Calculation”—as a faster completion method of computations. They used this approach for modelling and experimental validation of reliability in the pre-stressed masonry construction.

Research on the shear capacity of SBCW calculation by using optimised regression learner techniques (ORLTs) is limited. The only previous study that used regression techniques in SBCWs was that by Barakat et al. [21] in 2015. They proposed a model for predicting the shear buckling strength of SBCWs. The model calculation was based on the calculated interaction shear buckling of the specimen. They collected 93 experimental data from previous studies. These researchers concluded that the accurate prediction of the shear buckling strength of SBCWs was within a 95% confidence interval when minimal processing of data was performed.

The problem is that using some input parameters representing web dimensions and properties can contribute to a qualitatively higher level of the reliability assessment in computing the shear capacity of SBCWs. For this reason, the current study presents an alternative method for calculating the maximum shear capacity of SBCWs by using ORLTs through the known dimensions and steel grade of specimen webs. The model considers only web material properties and dimensions as major factors in the calculation without determining local, global and interaction shear buckling. The advantage of this model is that it requires extremely limited input data (i.e., web dimension and steel grade). In addition, the result obtained from the model parameters considers the interaction amongst various shear failure modes (i.e., local, global and interaction).

The objective of this research is to propose such a model that can predict the shear capacity of SBCWs computationally by using the steel grade and dimensions of a web determined from the preliminary design. The input data required for the model are web dimensions (thickness, height, shear span and corrugation geometric profile) and web yield stress. The resulting factor from the model represents the ratio of the web vertical shear stress to the normalised shear stress. The maximum shear capacity of the beam can be regarded as the model’s resulting factor multiplied by the normalised shear strength multiplied by the yielding vertical shear force. The research procedure can be summarised in seven steps. (1) Data relevant to more than 125 experimentally tested specimens are collected from the published studies of other researchers. (2) Test data and results from previous studies are organised in accordance with the corresponding parameters of the test specimens. (3) A summary of previously proposed formulas for predicting the SS of SBCWs with their corresponding theories is presented to identify the most dominant parameter that influences the SS of SBCWs. (4) The regression learner toolbox of MATLAB software is used for the regression process, which adopts four major optimised regression methods: decision tree (DT), support vector machines (SVM), Gaussian process regression (GPR) and ensemble trees (EN). Each regression method has different parameters obtained from the optimisation process. (5) The four ORLTs are tested by comparing the mean square error (MSE) and root-mean-square error (RMSE) calculated for each method. (6) In addition, another comparison is conducted between the results obtained from each of the four methods with that obtained from an artificial neural network (ANN) model. (7) Validation of the new modelling technique is achieved in two ways. Firstly, an experimental programme is conducted to test three specimens with different web geometries, steel grades and load setups. Secondly, the model results are compared with the test results obtained from two previous studies.

## 2. Theoretical Background

**Figure 1:**
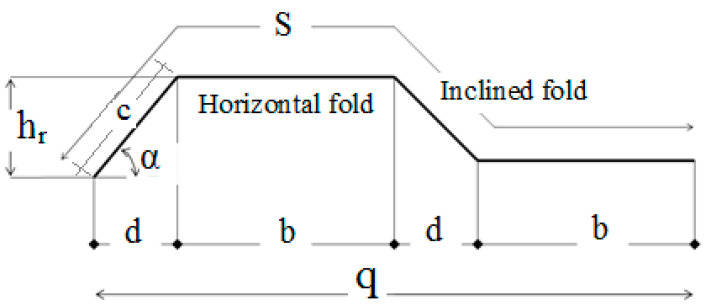
Corrugated web: profile configuration.

The local shear buckling stress of a corrugated web can be predicted in accordance with plate buckling theory [22]. Equation (1) expresses the corresponding local elastic shear buckling stress, $\tau_{L,el}$, on a single fold (longitudinal or inclined, Figure 1). In this case, each fold is assumed to be supported by the adjacent folds along its vertical edges and by the flanges along its horizontal edges.

(1) $$\tau_{el,\;L}=k_{L}\frac{\pi^{2}\;E}{12\left(1-v^{2}\right){\left(\omega /t_{W}\right)}^{2}}$$

where, *k_L_* is the local shear buckling coefficient that depends on the fold aspect ratio and the boundary conditions; *E* and *ν* are Young’s modulus and Poisson’s ratio, respectively; *w* is the fold width; *t_w_* is the web thickness. To determine the smallest value of *τ_L,el_*, *w* is set to be larger than c and b, as illustrated in Figure 1. Equation (1) has been used in many studies [1,2,4,5,6,23] to predict the local elastic shear buckling stress of corrugated webs.

On the basis of the expression for the global elastic shear buckling stress $\tau_{G,\;el}$ of corrugated plates proposed by Easley [24], Abbas [25] developed an equation to express the global shear buckling stress from geometric properties, i.e., Equation (2).

(2) $$\tau_{G,\;el}=k_{G}F\left(\alpha ,\;\beta \right)\frac{\;E\;t_{W}{}^{1/2}\;b^{3/2}}{12\;h_{w}^{2}}$$

where  F(α, β) is a relation between coefficient (*β*) and corrugation profile slope (*α*); (*β*) is a coefficient based on the relation between the ratio of b to c and the corrugation profile slope (*α*). This relation is proposed in Equation (3).

(3) $$F\left(\alpha ,\;\beta \right)=\sqrt{\frac{\left(1+\beta \right)\;\sin^{3}\alpha }{\beta +cos\;\alpha }}\;\left\{\frac{3\beta +1}{\beta^{2}\left(\beta +1\right)}\right\}$$

where, *h_w_* and *t_w_* are the web height and thickness, respectively.

To minimise *k_L_,* a small aspect ratio, *w*/*hw*, must be considered. In this case, *k_L_* lies between 5.34 and 8.98, assuming simply supported and fixed edges, respectively. In addition, a minimised *k_G_* can be obtained by assuming that the web is infinitely long [8]. By assuming that the web is long relative to *hw,* Elgaaly et al. [2] suggested in 1996 that *k_G_* should be set as 31.6 or 59 (assuming that the web is simply supported by flanges or flanges provide the web with fixed support, respectively). However, Easely [24] suggested in 1975 that *k_G_* varies between 36 and 68.4.

The general IB shear stress formula originally proposed by Lindner and Aschinger [1] is given in Equation (4).

(4) $$\frac{1}{{\left(\tau_{l,\;\mathrm{el}}\right)}^{n}}=\frac{1}{{\left(\tau_{L,\;\mathrm{el}}\right)}^{n}}+\frac{1}{{\left(\tau_{G,\;\mathrm{el}}\right)}^{n}}$$

where, *τ_I,el_*, *τ_L,el_* and *τ_G,el_* are interaction, local and global elastic shear buckling stresses.

Corresponding to Equation (4), Lindner and Aschinger [1] proposed two interaction formulas with *n* = 1 and *n* = 2. Yi et al. [6] used a formula that corresponds to Equation (4) with *n* = 1. Equation (4) is solved for *τ_I,el_*, as shown in Equation (5).

(5) $$\tau_{l,n,\;\mathrm{el}}=\frac{\tau_{L,\;\mathrm{el}}{\;\tau }_{G,\;\mathrm{el}}}{{\left[{\left(\tau_{L,\;\mathrm{el}}\right)}^{n}+{\left(\tau_{G,\;\mathrm{el}}\right)}^{n}\right]}^{1/n}}$$

On the basis of local, global and interaction buckling shear stresses, local, global and interaction buckling slenderness ratios can be, respectively, expressed as follows:

(6) $$\lambda_{L}=\sqrt{\frac{\tau_{y}}{\tau_{\mathrm{el},L}}}=\frac{\omega }{t_{w}}\sqrt{\frac{12\;\left(1-v^{2}\right){\;\tau }_{y}}{k_{L}\pi^{2}\;E}},$$

(7) $$\lambda_{G}=\sqrt{\frac{\tau_{y}}{\tau_{\mathrm{el},G}}}=\sqrt{\frac{12{\;\tau }_{y}{\;h}_{w}^{2}}{k_{G}\;F\left(\alpha ,\;\beta \right){\;E\;t}_{w}{}^{1/2}{\;b}^{3/2}\;}},$$

(8) $$\lambda_{l,\;n}=\sqrt{\frac{\tau_{y}}{\tau_{l,\;n,el}}}=\lambda_{L}\lambda_{G}{\left[{\left(1/\lambda_{L}\right)}^{2n}+{\left(1/\lambda_{G}\right)}^{2n}\right]}^{1/2n}.$$

where *λ_L_*, *λ_G_* and *λ_I,n_* are local, global and interaction buckling slenderness ratios; whereas, the *τ_y_* is shear yield stress, and *τ_I_* is the interaction shear buckling. Numerous studies have used these slenderness ratios to calculate the normalised local, global and interaction elastic shear buckling strength. The following formula was proposed by Yi et al. [6] for calculating normalised shear strength *(ρ_n,Y_*):

$$\rho_{n,\;Y}=\frac{\tau_{n,\;Y}}{\tau_{Y}}=1-0.614\;\left(\lambda_{l,1}-0.6\right)\leq 1.0\;if\;\lambda_{l,1}\leq \sqrt{2}\;,$$

(9) ρn, Y=τn, YτY=1(λl,1)2 if λl,1>2, 

where *ρ_n,Y_* is the normalised shear strength proposed by YI et al. [6]; *λ_l,_*_1_ is derived from Equation (8) with *n* = 1; $\tau_{Y}$ is the shear yield stress, which is equal to FY3.

## 3. Assessment of SBCW Shear Capacity Formulas

In accordance with previous studies and theories, maximum shear capacity can be largely determined from the contribution of the web. Therefore, the proposed formula is based on the calculated local, global and IB shear stresses for each specimen from Equations (6)–(8). Assume that web shear stress is constant over web height and equal to the average calculated shear stress. Hence, the web vertical shear stress can be calculated using Equation (10) as reported by [8].

(10) $$\tau =\frac{v_{n}}{h_{w}\;t_{w}},\;$$

where *V_n_* is the nominal vertical shear force in the steel beam.

Assume that (*ζ*) represents the ratio of the web vertical shear stress (*τ*) to the normalised shear stress (*τ_n,Y_*) as indicated in Equation (11) as reported by [8].

(11) $$\zeta =\frac{\tau }{\tau_{n,\;Y}}$$

Accordingly, from Equations (9)–(11), the following formula is proposed by the author to calculate the normalised shear force ($V_{n}$) (i.e., maximum shear capacity of a test specimen), and it is equal.

(12) $$V_{n}=\zeta \;\rho_{n,\;Y}\frac{F_{Y}}{\sqrt{3}}\;h_{w}\;t_{w}$$

where (*ρ_n, Y_*) is the normalised shear strength, and (*ζ*) is the ratio factor previously defined in Equation (11).

## 4. Test Data

The current study presents the database of 122 SS tests collected from 13 published studies [1,2,7,26,27,28,29,30,31]. This database is divided into two groups. The first group contains 115 published sources used in creating ORLT models. The second group consists of five shear tests collected from [32,33]. The group with three shear tests conducted by the authors is used to validate the model.

### 4.1. Test Data Published by Other Researchers

**Figure 2:**
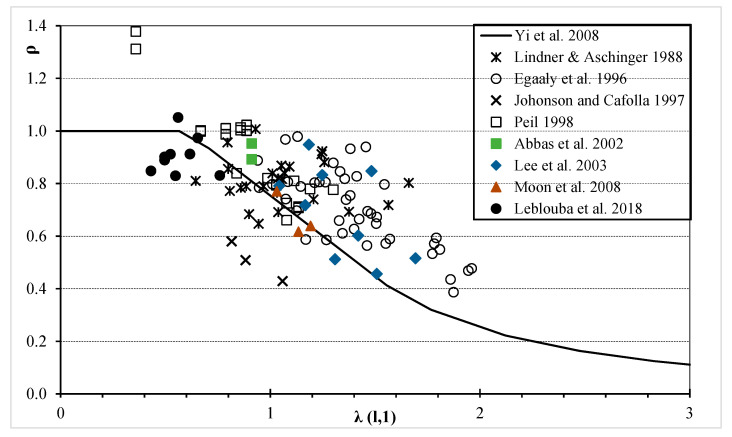
Relation between slenderness interaction shear buckling strength and normalised experimental shear strength.

The published test data are listed in Table A1, Table A2, Table A3, Table A4, Table A5, Table A6, Table A7, Table A8 and Table A9 (Appendix A). The dimensions of the test specimens are provided in Table A1, Table A2, Table A3, Table A4, Table A5, Table A6, Table A7, Table A8 and Table A9. These tests were conducted by the following authors. In 1996, Elgaaly et al. [2] reported the results of 42 tests (Table A1). The results of 25 tests from Sweden, Germany and Finland were reported by Lindner and Aschinger [1] in 1998 (Table A2). Johnson and Cafolla [26] summarised the results of three specimens in 1997 (Table A3). The results of 20 specimens were tested under shear forces by Peil [27] in 1998 (Table A4). Driver et al. [5] presented the shear test results of two steel girders with corrugated webs in 2002 (Table A5). Lee et al. [28] reported the results of nine shear tests in 2003 (Table A6). Moon et al. [7] summarised the results of three shear tests in 2008 (Table A7). Moussa et al. [29] provided the results of nine tests in 2018 (Table A8). Wang et al. [30], Sause and Clarke [23] and Hannebauer et al. [31] reported the results of one test each in 2019 (Table A9). In these tables, the definitions of symbols *h_w_, b_p_, t_w_, b, d, h_r_* and *F_y_* are the same as those given earlier; whereas *h_w_/t_w_* is the web slenderness. From a previous study [6], a conclusion is drawn that the normalised SS exhibits an indirect relation with the slenderness interaction shear buckling strength. Therefore, for all the test specimens listed in Table A1, Table A2, Table A3, Table A4, Table A5, Table A6, Table A7, Table A8 and Table A9, Figure 2 shows the normalised experimental SS $\rho_{e}=\tau_{e}/\tau_{y}$ versus the interaction slenderness ratio at n = 1 (*λ_I,_*_1_). The comparison between the normalised SS proposed by Yi et al. [6] (*ρ_n,Y_*) and the normalised experimental SS is illustrated in Figure 2. The horizontal axis in this figure represents the slenderness interaction shear buckling strength with the exponent n = 1. As shown in this figure, the major factors that affect shear capacity are web height, web panel, web thickness, corrugation geometry and web yield stress.

### 4.2. Test Data from the Authors

**Figure 3:**
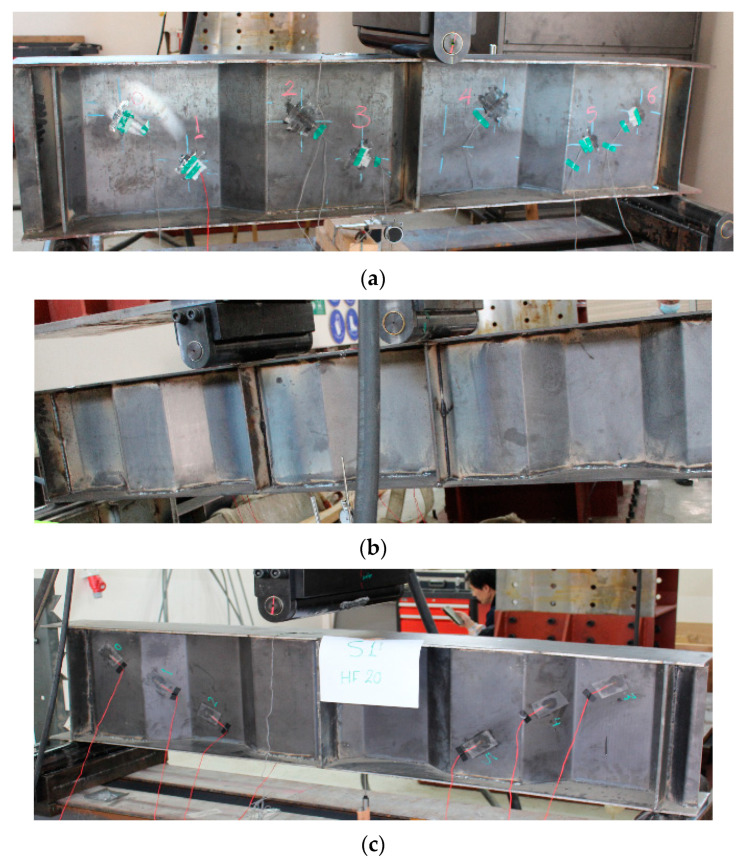
Sample of SBCW specimens: (**a**) Specimen 3PCW350, (**b**) Specimen 4PCW275 and (**c**) Specimen 3PCW200.

To validate the model, a series of three tests were conducted on SBCWs with different properties, dimensions and load cases (Figure 3). The load cases and dimensions of the test beams, which are denoted as 3PCW350, 4PCW275 and 3PCW200, and the material yield strength are provided in Table 1. In this table, ‘P’ and ‘CW’ represent ‘point load’ and ‘corrugated web,’ respectively; the number before ‘P’ represents the number of line loads applied. Meanwhile, the number following ‘CW’ indicates horizontal fold (HF) length (in mm). All the specimens were simply supported and loaded on a hydraulic testing machine by applying displacement control techniques at the civil engineering laboratories of Taif University. Specimens 3PCW350 and 3PCW200 have an HF of 350 mm and 200 mm, respectively. The two specimens have the same web yield stress and tested under a three-point load, as shown in Figure 3a,c. Specimen 4PCW275 has an HF of 275 mm and different yield stresses. It was tested under a four-point load as shown in Figure 3b. The three specimens analogised one another in the inclined fold dimensions and corrugation angle.

### 4.3. Test Setup

**Figure 4:**
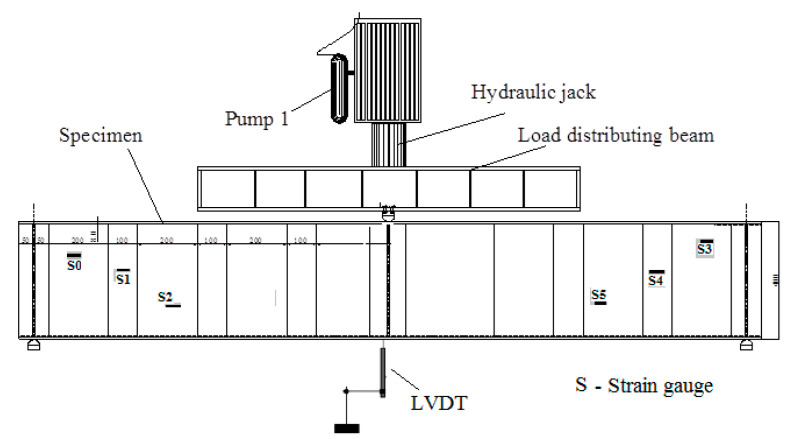
Test scheme.

The specimens were tested at Taif University in Saudi Arabia by using a 2000 kN capacity test frame, as shown in Figure 4. The specimens were tested under different loading conditions (three- and four-line loads). The unbraced length of the compressive flange was 1800 mm and 2250 mm for the three- and four-line loads, respectively, in accordance with the locations of the supports. The total length of a specimen was longer than the unbraced length of the compression flange by 100 mm (50 mm from each side). The shear span for the three-line loads was 900 mm, and for the four-line loads was 750 mm. The primary objective for fabricating and testing the specimens was to validate the proposed model under variable parameters and not to compare it with each other. The parameters were different for each specimen, such as HF length, loading type, shear span, web thickness and web yield stress. The specimens were loaded using displacement control techniques with an increment of 0.005 mm/s. To measure the vertical deflections of the specimens, a linear variable differential transformer (LVDT) was installed under the mid-span of each specimen, as illustrated in Figure 4.

**Figure 5:**
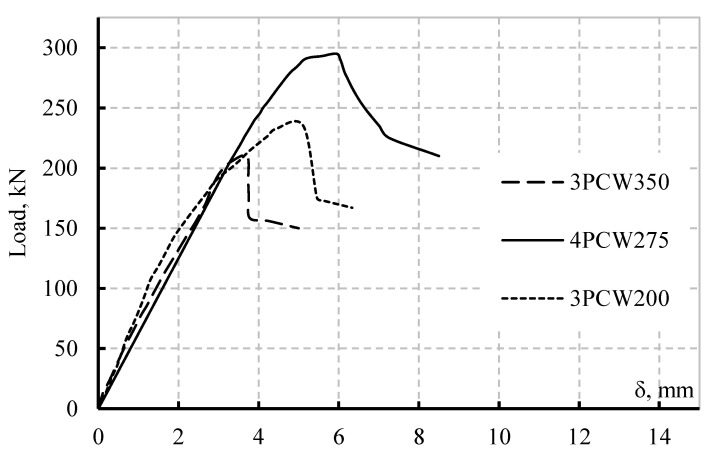
Load deflection curves of the specimens.

The fabricated specimens had the same flange material properties and dimensions, whilst the webs had different material and geometric (HF length and thickness) properties. For all the specimens, the web slenderness ratio belongs to Class 4 in accordance with the Eurocodes. From the test results, the plot of the vertical load versus the mid-span vertical deflection of each tested specimen is shown in Figure 5. The maximum deflections achieved by the three-line load specimens (3PCW200 and 3PCW350) were 3.8 mm and 5 mm, respectively, before failure. Meanwhile, the maximum deflection recorded for specimen 4PCW275 was nearly 6 mm. Specimen 4PCW275 exhibited lower initial stiffness than the two specimens subjected to three-line loads. Conversely, the maximum shear force sustained by Specimen 4PCW275 (147.50 kN) was higher than those of the three-line load specimens, i.e., 3PCW200 (117.5 kN) and 3PCW350 (105 kN). Such difference is attributed to the four-line load specimen having higher web thickness and web yield stress than the three-line load specimens. This result is reasonable because web thickness and web yield stress are the most dominant parameters that influence the shear capacity of SBCWs.

## 5. ORLTs

The regression learner toolbox of MATLAB software is one of the most frequently used techniques for regression. It has four major optimised regression methods [34]: DT, SVM, GPR and EN. Each regression has different parameters obtained from the optimisation process. For example, the SVM hyperparameter search range is selected as follows: box constraints varied from 0.001 to 1000; the kernel scale varied from 0.001 to 1000; epsilon varied from 0.00030022 to 30.0222; the kernel functions were Gaussian, linear, quadratic, and cubic; the standardised data were true and false [34]. The optimal parameters of each ORLT were determined and evaluated based on the Bayesian optimisation (BO) technique [35,36,37]. The acquisition function used in the optimisation process was an expected improvement per second plus, and the total number of iterations was 30. The BO technique is the most effective approach used to determine the hyperparameters of the ORLTs during the training stage [35]. The BO technique determines the optimal parameters of each regression technique during each training step based on the prior and the probability space value of each parameter and choosing the highest probability values used to enhance the predicting accuracy of the ORLT model [36]. The details determining the best parameters of each regression technique using the BO technique were presented in [36] and [37].

The input features of the dataset samples were firstly normalised before the training process, as follows:

(13) $$x_{i}=\frac{x_{i}-MIN_{i}}{MAX_{i}-MIN_{i}},\;i=1,\;2,,\;....,\;8,\;$$

where *x_i_* is the *i*th input feature; *MIN_i_* and *MAX_i_* are the minimum and maximum values of the *i*th input feature, respectively.

**Figure 6:**
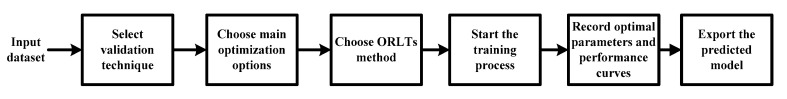
Training procedure of ORLTs.

Figure 6 introduces the training procedure of ORLTs by using MATLAB’s regression learner toolbox in 2020b MATLAB/Software.

The training procedure of the ORLTs can be summarised as follows:

1. The validation technique was selected, the cross-validation technique with 10 folds was chosen before the training process.
2. The primary optimisation options were selected, and the option used was the BO technique, with an expected improvement per second plus and 30 iterations.
3. One of the ORLTs was selected (DT, SVM, GPR or EN).
4. The training process is started to determine the optimal parameters and predicted model of this method.
5. The optimal parameters and performance model of the selected method were recorded.
6. Finally, the ORLT model of the selected method was exported to be used in the prediction of the original and new datasets.

**Figure 7:**
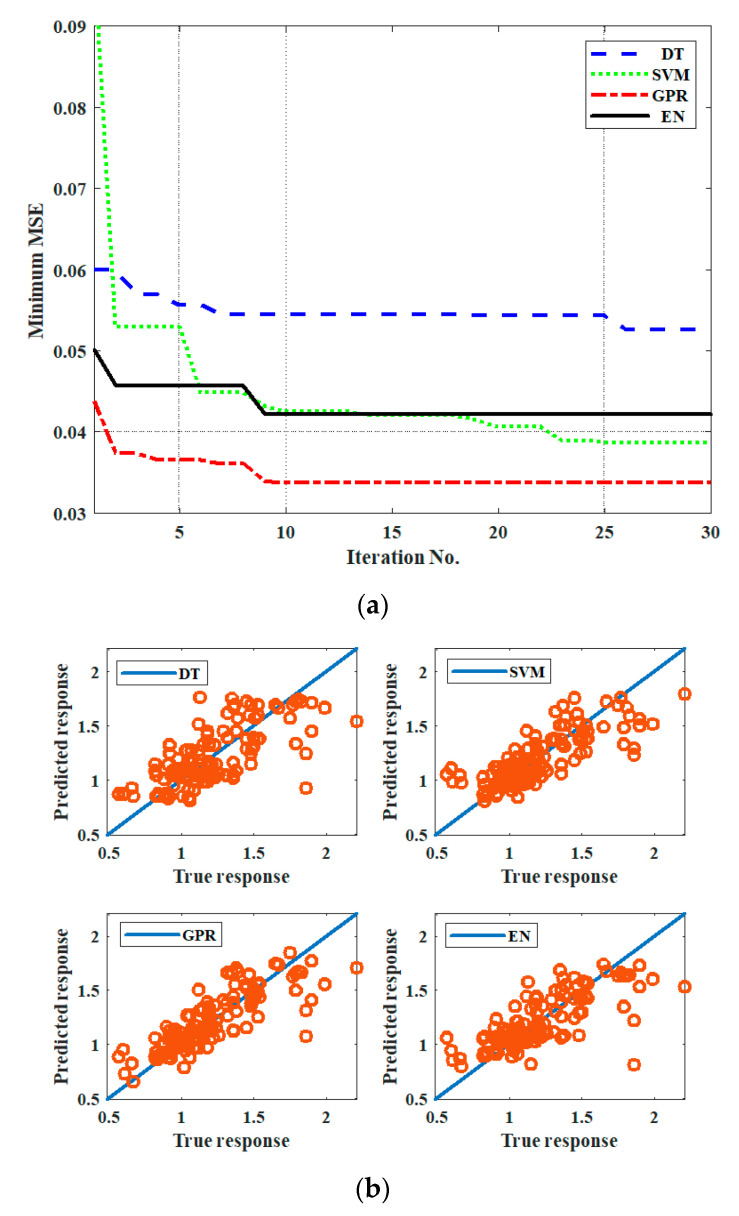
(**a**) Minimum MSE vs. iteration number; (**b**) predicted response vs. true response during training.

Figure 7a presents the minimum MSE versus the training iteration numbers of DT, SVM, GPR and EN during training. The MSE values of the four regression methods illustrated that GPR achieved the lowest MSE amongst the four methods. Meanwhile, DT exhibited poor training performance. Figure 7b illustrates the relation between the predicted responses versus the true responses of the four regression methods. GPR exhibited the best response amongst the four methods.

Table 2 provides the optimal parameters of the four ORLT models. For example, the optimal parameters of the EN model are as follows: the selected ensemble method is the bag; the number of learners is 57; the minimum leaf size is 2; the number of predictors to samples is 8. The optimal parameters of the DT, SVM and GPR models are listed in Table 2.

## 6. Model Validation and Comparison

**Figure 8:**
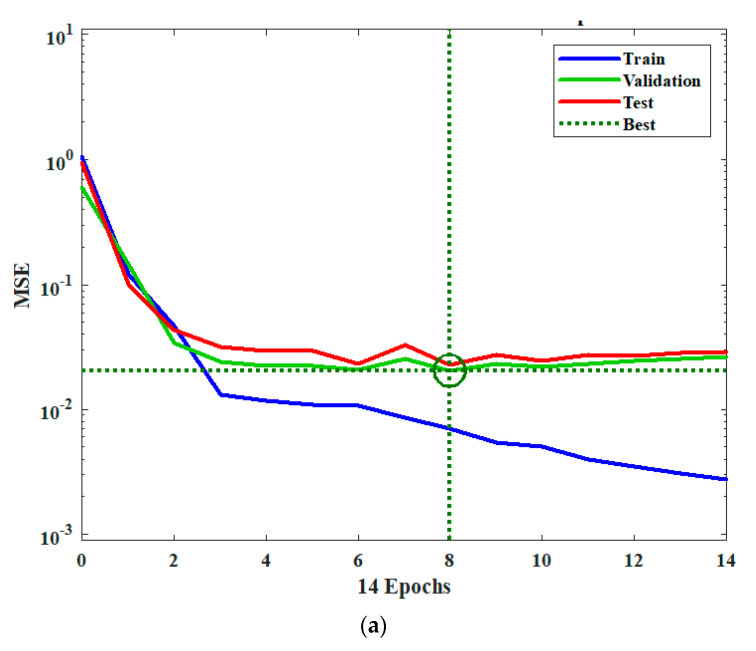

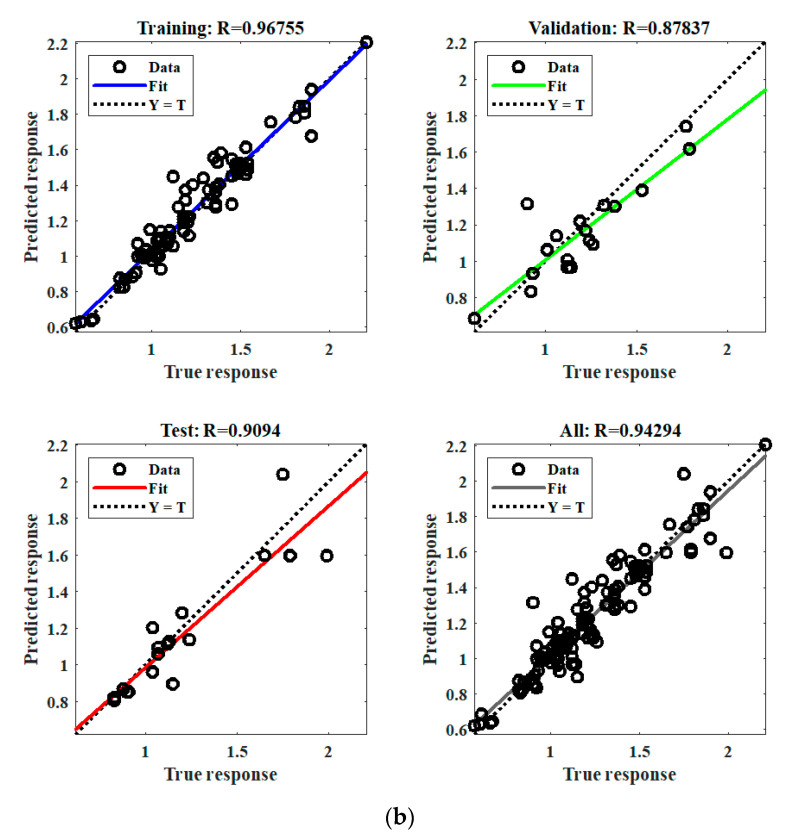
Validation and testing sets: (**a**) MSE vs. iteration epochs and (**b**) predicted response vs. true response.

The four ORLTs were tested and validated by comparing the calculated ratio for each specimen (ratio of web vertical shear stress to normalised shear stress) with that obtained from an ANN. MATLAB’s ANN toolbox was used to train and test the ANN model. The normalised dataset (120 samples) was used as input for the ANN, and the corresponding ratio of web vertical shear stress (*τ*) to normalised shear stress (*τ_n,Y_*) (Equation (11)) was used as the output for the training stage of the ANN. The ANN model consisted of three layers: the input, hidden and output layers. The number of neurons in the input layer was equal to the number of input features (eight input layers). The number of neurons in the hidden layer was selected to enhance the performance of the ANN model (24 neurons were used here). The number of output layers is equal to the number of output variables (one layer is used here). The normalised 120 dataset samples were divided during the training stage of the ANN model into three sets: for training (84 samples, 70%), validation (18 samples, 15%) and testing (18 samples, 15%). The MSE performance of the ANN model and the predicted responses for the training, validation and testing sets are presented in Figure 8.

Table 3 provides the MSE and RMSE of the differences between the values of *ζ* estimated using the model and calculated theoretically from the test database using Equation (11). From the results in Table 3, the authors concluded that GPR is the most suitable and accurate method for estimating the ratio *ζ* with an acceptable degree of accuracy.

## 7. Initial Comparison with Published Experimental Data

The analysis of the test results of the test specimens is presented in Appendix A (Table A10, Table A11, Table A12, Table A13, Table A14, Table A15, Table A16, Table A17 and Table A18). A specimen identifier is given in the first column, and the local slenderness ratio is provided in the second column. The third column presents the global slenderness ratio, and the fourth column shows the interaction slenderness ratio *λ_I,_*_1_ [Equation (8), with n = 1]. The fifth column provides the normalised SS from Equation (9), *ρ_n,Y_*. The sixth column lists the values of *ζ* calculated using GPR. The seventh column gives the anticipated maximum shear force (*V_n_*) by using Equation (12), whilst the eighth column provides the shear test results (*V_T_*). The last column indicates the ratio of *V_n_* to *V_T_*.

Table 4 provides a summary of the results divided into three groups. The first group represents the results of 76 specimens. The number of specimens in this group is equivalent to 65% of the total number, and the results of shear forces from the proposed model are between ±1% of the test shear results. The second group represents the results of 38 specimens, which is equivalent to 32% of the total number of collected specimens. The maximum shear force calculated in Group 2 by using the proposed model is ±5% of the corresponding specimen’s test shear results. The last group included three specimens, which is equivalent to nearly 3% of the total. The maximum shear forces anticipated by the model exhibit −8% to 10% of the corresponding specimen’s test shear results.

To validate the proposed model, the database of six tests from two previous studies and three tests conducted by the authors were presented in this paper. Table 5 provides the dimensions of the test specimens from the following studies: Moussa et al. [32] reported the results of four tests, and Nie et al. [33] summarised the results of two specimens. The database of the three tests conducted by the authors is presented in Table 1.

By using the preceding data as a database for the proposed model, the maximum capacity of the shear force that can be resisted by each specimen is provided in Table 6. The ratio of the model results to the experimental results is ±9%.

## 8. Conclusions

This study presented a new approach for calculating the maximum shear capacity of SBCWs. The approach was implemented using ORLTs. Four regression methods were used to select the most appropriate one, which could achieve the least MSE. The model was created on the basis of 125 test results of different specimen parameters obtained by previous researchers. The input parameters were the web dimensions (thickness, height, shear span and corrugation geometric profile) and web yield stress. The model output was the ratio of the web vertical shear stress to the normalised shear stress. Validation of the model results was determined using both an experimental programme conducted by the authors and an experimental database from previous studies. The following conclusions can be drawn from the obtained results: (i) The model procedures to calculate the maximum shear capacity of steel beams with corrugated web are well-suited for the design of beam elements in load-carrying with the required level of reliability. (ii) The shear capacity of SBCWs can be predicted to an acceptable degree of accuracy by using the resulting factor from the proposed model. (iii) The proposed model exhibited a percentage error on the shear capacity of less than ±5% for 97% of the total number of specimens. (iv) ORLTs methods can be used in calculating the design shear of SBCWs. (v) The most appropriate method for calculating the shear force of SBCWs is the GPR method. (vi) The mean square error (MSE), as the difference between the resulting output factors and those calculated for each specimen, was less than 0.1%.

## Figures and Tables

**Table 1:** Test data from the authors.

Specimen ID	*h_w_* (mm)	a (mm)	*t_w_* (mm)	Corrugation Dimensions (mm)			*F_yw_* MPa	Web Slenderness	Variables
				*b*	*h_r_*	*d*			
3PCW350	384	900	2.80	350	100	100	325	137.14	*b*, Load
4PCW275	384	750	3.00	275	100	100	357	128.00	*a,t_w_, F_yw_*
3PCW200	384	900	2.80	200	100	100	325	137.14	*b*, Load

**Table 2:** Optimal parameters of each ORLT.

ORLTs	Optimal Parameters
DT	Minimum leaf size: 12
SVM	Box constraints: 992.584 Epsilon: 0.00031804 Kernel function: Linear Standardised data: False
GPR	Sigma: 0.16623 Basis function: Zero Kernel function: Isotropic exponential Kernel scale: 0.66404 Standardised data: True
EN	Ensemble method: Bag Number of learners: 57 Minimum leaf size: 2 Number of predictors to samples: 8

**Table 3:** Comparison of the ORLT models with the ANN model.

Evaluation Techniques	DT	SVM	GPR	EN	ANN
MSE	0.04212	0.03962	0.00074	0.00391	0.01088
RMSE	0.20522	0.19906	0.02723	0.06253	0.10432

**Table 4:** Mean, standard deviation (Std. dev.), coefficient of variation (Co. Var.), maximum (Max.) and minimum (Min).

Number of Test Data	Mean	Std. Dev.	Co. Var.	Max.	Min.
116	1.0018	0.021	0.021	1.10	0.926
76 out of 116 (65%)	0.987	0.015	0.015	1.015	0.986
38 out of 116 (32%)	1.0033	0.029	0.028	1.055	0.955
3 out of 116 (3%)	1.0185	0.071	0.07	1.10	0.926

**Table 5:** Data of specimens tested in previous studies.

Specimen ID	*h_w_*	a	*t_w_*	Corrugation Dimensions			*F_yw_*	Web Slenderness
				*b*	*h_r_*	*d*		
Moussa et al. [32]								
A12-305-30	305	557.0	1.20	40	20.00	34.64	230	254.17
A12-410-30	410	557.0	1.20	40	20.00	34.64	230	341.67
A12-505-30	505	557.0	1.20	40	20.00	34.64	230	420.83
A12-505-45	505	526.5	1.20	40	28.28	28.28	230	420.83
Nie et al. [33]								
S2-1	260	1200	0.90	80	48	64	385.50	288.89
S2-2	360	1200	0.90	80	48	64	385.50	400.00

**Table 6:** Analysis results of test specimens reported in previous studies.

Specimen ID	*λ_L_*	*λ_G_*	*λ_I,1_*	*ρ _e_*	*ζ*	*V_n_* (kN)	*V_T_* (kN)	*V_n_/V_T_*
Moussa et al. [32]								
A12-305-30	0.391	0.34	0.52	1.00	0.94	45.60	49.8	0.92
A12-410-30	0.391	0.46	0.60	1.00	0.98	64.21	66.3	0.97
A12-505-30	0.391	0.56	0.69	0.95	1.03	78.46	72	1.09
A12-505-45	0.391	0.43	0.58	1.00	1.03	82.99	89.1	0.93
Nie et al. [33]								
S2-1	1.349	0.21	1.37	0.53	0.90	24.88	25.24	0.99
S2-2	1.349	0.29	1.38	0.52	0.97	36.58	39.31	0.93
Authors								
3PCW350	1.74	0.16	1.75	0.327	1.56	102.94	105.00	0.98
4PCW275	1.339	0.15	1.35	0.54	1.16	149.36	147.50	1.01
3PCW200	0.995	0.13	1.00	0.752	0.80	121.45	117.50	1.03

## Data Availability

The data presented in this study are available on request from the corresponding author.

## Appendix A. Experimental Data

**Table A1:** Data of specimens tested by Lindner and Aschinger [1].

Specimen ID	*h_w_*	*a*	*t_w_*	Corrugation Dimensions			*F_yw_*	Web Slenderness
				*b*	*h_r_*	*d*		
L1A	994	974	2	140	50.03	50	292	512.37
L1B	994	984	3	140	50.03	50	335	383.78
L2A	1445	1503	2	140	50.03	50	282	744.85
L2B	1445	1503	3	140	50.03	50	317	568.90
L3A	2005	2005	2	140	50.03	50	280	997.51
L3B	2005	2005	3	140	50.03	50	300	792.49
B1	600	798	2	140	50.03	50	341	285.71
B4	600	798	2	140	50.03	50	363	284.36
B4b	600	798	2	140	50.03	50	363	284.36
B3	600	798	3	140	50.03	50	317	229.01
B2	600	702	3	140	50.03	50	315	229.01
M101	600	600	1	70	15.01	15	189	606.06
M102	800	800	1	70	15.01	15	190	808.08
M103	1000	1000	1	70	15.01	15	213	1052.63
M104	1200	1200	1	70	15.01	15	189	1212.12
L1	1000	1500	2	106	50.02	87	410	476.19
L1	1000	1490	3	106	50.02	87	450	333.33
L2	1498	2157	2	106	50.02	87	376	749.00
L2	1498	2142	3	106	50.02	87	402	499.33
No. 1	850	1131	2	102	55.55	86	355	425.00
No. 2	850	1131	2	91	56.30	72	349	425.00
V1/1	298	2819	2	144	102.06	102	298	145.37
V1/2	298	2000	2	144	102.06	102	283	141.90
V1/3	298	1001	2	144	102.06	102	298	149.00
V2/3	600	1650	3	144	102.06	102	279	200.00

**Table A2:** Data of specimens tested by Elgaaly et al. [2].

Specimen ID	*h_w_*	*a*	*t_w_*	Corrugation Dimensions			*F_yw_*	Web Slenderness
				*b*	*h_r_*	*d*		
V-PILOTA	305	305	0.78	38.10	25.42	25.40	621	390.03
V-PILOTB	305	305	0.79	38.10	25.42	25.40	638	388.54
V121216A	305	305	0.64	38.10	25.42	25.40	676	478.06
V121216B	305	305	0.77	38.10	25.42	25.40	665	398.69
V181216B	457	305	0.61	38.10	25.42	25.40	618	749.18
V181216C	457	305	0.76	38.10	25.42	25.40	679	602.11
V181816A	457	457	0.64	38.10	25.42	25.40	591	719.69
V181816B	457	457	0.74	38.10	25.42	25.40	614	620.08
V241216A	610	305	0.64	38.10	25.42	25.40	591	960.63
V241216B	610	305	0.79	38.10	25.42	25.40	588	775.10
V121221A	305	305	0.63	41.90	33.45	23.40	665	484.13
V121221B	305	305.00	0.79	41.90	33.45	23.40	665	388.54
V122421A	305	609.60	0.68	41.90	33.45	23.40	621	451.18
V122421B	305	609.60	0.78	41.90	33.45	23.40	638	390.03
V181221A	457	305	0.61	41.90	33.45	23.40	578	749.18
V181221B	457	305	0.76	41.90	33.45	23.40	606	599.74
V181821A	457	457	0.64	41.90	33.45	23.40	552	719.69
V181821B	457	457	0.74	41.90	33.45	23.40	596	620.08
V241221A	610	305	0.61	41.90	33.45	23.40	610	1000.00
V241221B	610	305	0.76	41.90	33.45	23.40	639	800.52
V121232A	305	305	0.64	49.80	50.77	26.40	665	476.56
V121232B	305	305	0.78	49.80	50.77	26.40	641	391.03
V121832A	305	457	0.64	49.80	50.77	26.40	703	476.56
V121832B	305	457	0.92	49.80	50.77	26.40	562	331.88
V122432A	305	609.60	0.64	49.80	50.77	26.40	714	476.56
V122432B	305	609.60	0.78	49.80	50.77	26.40	634	392.54
V181232A	457	305	0.60	49.80	50.77	26.40	552	765.49
V181232B	457	305	0.75	49.80	50.77	26.40	602	610.15
V181832A	457	457	0.61	49.80	50.77	26.40	689	749.18
V181832B	457	457	0.75	49.80	50.77	26.40	580	610.15
V241232A	610	305.00	0.62	49.80	50.77	26.40	673	980.71
V241232B	610	305.00	0.76	49.80	50.77	26.40	584	800.52
V121809A	305	457.00	0.71	19.80	14.19	11.90	572	432.01
V121809C	305	457.00	0.63	19.80	14.19	11.90	669	482.59
V122409A	305	609.60	0.71	19.80	14.19	11.90	586	427.17
V122409C	305	609.60	0.66	19.80	14.19	11.90	621	460.03
V181209A	457	305.00	0.56	19.80	14.19	11.90	689	817.53
V181209C	457	305.00	0.61	19.80	14.19	11.90	592	749.18
V181809A	457	457.00	0.61	19.80	14.19	11.90	618	749.18
V181809C	457	457.00	0.62	19.80	14.19	11.90	559	734.73
V241209A	610	305.00	0.62	19.80	14.19	11.90	606	980.71
V241209C	610	305.00	0.64	19.80	14.19	11.90	621	960.63

**Table A3:** Data of specimens tested by Johnson and Cafolla [26].

Specimen ID	*h_w_*	*a*	*t_w_*	Corrugation Dimensions			*F_yw_*	Web Slenderness
				*b*	*h_r_*	*d*		
CW1	440.36	730.92	3.06	180	45.01	44.99	320	143.91
CW2	437.92	730.92	3.29	180	45.01	44.99	312	133.11
CW3	437.18	940.92	3.26	250	45.01	44.99	284	134.10

**Table A4:** Data of specimens tested by Peil [27].

Specimen ID	*h_w_*	*a*	*t_w_*	Corrugation Dimensions			*F_yw_*	Web Slenderness
				*b*	*h_r_*	*d*		
SP1	800	1750	2	146	104.07	104	307	400
SP2	800	1750	2	170	80.05	80	299	400
SP3	800	1750	2	185	65.04	65	292	400
SP4	800	1800	2	117	83.05	83	298	400
SP5	800	1800	2	136	64.04	64	291	400
SP6	800	1800	2	148	52.03	52	294	400
SP2-2-400 1	400	1000	2	170	80.05	80	263	200
SP2-2-400 2	400	1000	2	170	80.05	80	263	200
SP2-2-800 1	800	1000	2	170	80.05	80	272	400
SP2-2-800 2	800	1000	2	170	80.05	80	272	400
SP2-3-600 1	600	1000	3	170	80.05	80	294	200
SP2-3-600 2	600	1000	3	170	80.05	80	294	200
SP2-3-1200 1	1200	1000	3	170	80.05	80	294	400
SP2-3-1200 2	1200	1000	3	170	80.05	80	294	400
SP2-4-800 1	800	1000	4	170	80.05	80	326	200
SP2-4-800 2	800	1000	4	170	80.05	80	326	200
SP2-4-1600 1	1600	1000	4	170	80.05	80	328	400
SP2-4-1600 2	1600	1000	4	170	80.05	80	328	400
SP2-8-800 1	800	1000	8	170	80.05	80	270	100
SP2-8-800 2	800	1000	8	170	80.05	80	270	100

**Table A5:** Data of specimens tested by Driver et al. [5].

Specimen ID	*h_w_*	*a*	*t_w_*	Corrugation Dimensions			*F_yw_*	Web Slenderness
				*b*	*h_r_*	*d*		
G7A	1500	4500	6	300	150	200	465	250
G8A	1500	4500	6	300	150	200	465	250

**Table A6:** Data of specimens tested by Lee et al. [28].

Specimen ID	*h_w_*	*a*	*t_w_*	Corrugation Dimensions			*F_yw_*	Web Slenderness
				*b*	*h_r_*	*d*		
L1	1500	3000	4.80	450	200.00	300	250	312.50
L2	1500	3400	4.80	550	188.80	300	250	312.50
L3	1500	3000	4.80	450	49.60	300	250	312.50
L4	1500	3400	4.80	550	55.60	300	250	312.50
I1	2000	3600	4.80	320	44.60	100	250	416.67
I2	2000	3600	3.80	350	28.60	100	250	526.32
G1	2000	3000	4.80	200	45.40	180	250	416.67
G2	2000	3000	3.80	160	33.00	50	250	526.32
G3	2000	3000	3.80	160	26.90	100	250	526.32

**Table A7:** Data of specimens tested by Moon et al. [7].

Specimen ID	*h_w_*	*a*	*t_w_*	Corrugation Dimensions			*F_yw_*	Web Slenderness
				*b*	*h_r_*	*d*		
PG2	2000	2600	4	250	60	220	296	500
PG1	2000	2800	4	220	60	180	296	500
PG3	2000	2800	4	220	75	180	296	500

**Table A8:** Data of specimens tested by Moussa et al. [29].

Specimen ID	*h_w_*	*a*	*t_w_*	Corrugation Dimensions			*F_yw_*	Web Slenderness
				*b*	*h_r_*	*d*		
TP20-300-30	3050	664.90	2	60	20.01	34.64	290	152.50
A20-410-30-N	410	578.10	2	40	20.01	34.64	290	205.00
A20-410-45-N	410	524.80	2	40	28.29	28.28	290	205.00
A20-505-30-N	505	575.70	2	40	20.01	34.64	290	252.50
A20-505-45-N	505	525.20	2	40	28.29	28.28	290	252.50
B20-305-30	305	427.00	2	40	20.01	34.64	680	152.50
B20-305-45	305	390.40	2	40	28.29	28.28	680	152.50
B20-505-45	505	388.85	2	40	28.29	28.28	680	252.50
B20-505-45-N	505	388.85	2	40	28.29	28.28	290	252.50

**Table A9:** Test data reported by Wang et al. [30], Sause [8] and Hannebauer [31].

Specimen ID	*h_w_*	*a*	*t_w_*	Corrugation Dimensions			*F_yw_*	Web Slenderness
				*b*	*h_r_*	*d*		
W1	1200	2000	3	110	55	90	400	400
SC1	1500	4500	6.27	300	150	200	465	239.23
V1b	500	1000	2.50	30	40	47	270	200

**Table A10:** Analysis results of test specimens reported by Lindner and Aschinger [1].

Specimen ID	*λ_L_*	*λ_G_*	*λ_I,1_*	*ρ _e_*	*ζ*	*V* (kN)	*V_T_* (kN)	*V/V_T_*
L1A	0.953	0.53	1.09	0.70	1.255	281.72	280.93	1.01
L1B	0.765	0.53	0.93	0.80	1.238	495.08	501.34	0.98
L2A	0.937	0.76	1.21	0.63	1.222	347.69	337.50	1.04
L2B	0.759	0.75	1.07	0.71	1.182	571.69	563.51	1.00
L3A	0.901	1.04	1.38	0.52	1.384	465.59	451.22	1.05
L3B	0.741	1.02	1.26	0.60	1.460	776.65	773.57	0.99
B1	0.952	0.34	1.01	0.75	1.056	202.53	208.04	0.94
B4	0.977	0.35	1.04	0.73	1.062	203.28	183.46	1.12
B4b	0.977	0.35	1.04	0.73	1.062	203.28	217.66	0.95
B3	0.735	0.31	0.80	0.88	1.008	255.36	246.01	1.04
B2	0.733	0.31	0.80	0.88	1.061	263.45	273.42	0.98
M101	0.751	0.74	1.05	0.72	1.153	53.46	52.96	1.02
M102	0.753	0.99	1.24	0.60	1.507	79.10	79.19	1.00
M103	0.831	1.32	1.56	0.41	1.751	83.79	83.95	1.00
M104	0.751	1.48	1.66	0.36	2.168	102.33	103.98	0.98
L1	0.790	0.66	1.03	0.74	1.045	383.35	380.08	1.01
L1	0.579	0.63	0.86	0.84	0.945	617.09	610.72	1.01
L2	0.794	0.96	1.25	0.60	1.506	592.39	600.20	0.98
L2	0.548	0.90	1.05	0.72	1.202	899.87	905.32	1.00
No. 1	0.743	0.48	0.88	0.83	0.948	272.59	275.01	0.99
No. 2	0.657	0.47	0.81	0.87	0.918	272.46	264.27	1.04
V1/1	0.939	0.10	0.94	0.79	0.820	67.96	67.98	1.00
V1/2	0.894	0.09	0.90	0.82	0.837	69.87	69.84	1.00
V1/3	0.963	0.10	0.97	0.77	0.992	79.08	80.93	0.97
V2/3	0.621	0.17	0.64	0.97	0.844	236.78	234.89	1.01

**Table A11:** Analysis result of test specimens reported by Elgaaly et al. [2].

Specimen ID	*λ_L_*	*λ_G_*	*λ_I,1_*	*ρ _e_*	*ζ*	*V* (kN)	*V_T_* (kN)	*V/V_T_*
V-PILOTA	0.939	0.52	1.07	0.71	1.284	76.65	82.73	0.94
V-PILOTB	0.948	0.52	1.08	0.70	1.290	78.78	71.17	1.12
V121216A	1.200	0.57	1.33	0.55	1.249	50.51	50.05	1.05
V121216B	0.993	0.54	1.13	0.67	1.300	80.36	87.63	0.90
V181216B	1.200	0.82	1.46	0.47	1.870	89.05	93.41	0.94
V181216C	1.011	0.82	1.30	0.57	1.537	117.97	119.47	1.00
V181816A	1.128	0.80	1.38	0.52	1.492	75.66	74.73	1.03
V181816B	0.990	0.78	1.26	0.59	1.370	96.48	96.17	1.01
V241216A	1.128	1.06	1.55	0.42	1.423	77.15	75.57	1.04
V241216B	0.908	1.01	1.35	0.54	1.494	133.19	133.35	0.98
V121221A	1.326	0.45	1.40	0.51	1.238	47.01	46.26	1.01
V121221B	1.064	0.42	1.15	0.67	1.188	75.75	72.50	1.00
V122421A	1.194	0.42	1.27	0.59	1.034	44.47	43.28	1.04
V122421B	1.046	0.41	1.13	0.68	1.017	61.61	61.20	0.99
V181221A	1.277	0.63	1.42	0.49	1.380	64.04	61.83	1.02
V181221B	1.046	0.61	1.21	0.62	1.301	99.89	97.86	1.01
V181821A	1.198	0.61	1.34	0.54	1.208	58.44	56.49	1.07
V181821B	1.073	0.61	1.23	0.61	1.269	92.84	93.41	0.96
V241221A	1.312	0.86	1.57	0.41	1.481	78.46	77.26	1.02
V241221B	1.075	0.84	1.36	0.53	1.403	130.66	126.72	1.01
V121232A	1.782	0.30	1.81	0.31	1.702	39.58	41.14	0.95
V121232B	1.436	0.28	1.46	0.47	1.460	59.92	61.16	0.98
V121832A	1.833	0.31	1.86	0.29	1.493	34.40	34.47	0.99
V121832B	1.141	0.25	1.17	0.65	0.994	54.68	53.38	1.10
V122432A	1.847	0.31	1.87	0.28	1.360	31.35	31.14	1.00
V122432B	1.434	0.28	1.46	0.47	1.175	49.14	48.93	0.98
V181232A	1.741	0.42	1.79	0.31	1.848	49.76	51.60	0.97
V181232B	1.449	0.41	1.51	0.44	1.536	79.59	80.06	1.00
V181832A	1.904	0.47	1.96	0.26	1.810	52.07	52.93	0.99
V181832B	0.939	0.52	1.07	0.71	1.284	76.65	82.73	0.94
V241232A	1.422	0.41	1.48	0.46	1.505	78.12	78.64	1.00
V241232B	1.845	0.61	1.94	0.26	1.765	69.16	69.08	1.00
V121809A	1.403	0.54	1.50	0.44	1.485	103.31	101.46	1.01
V121809C	0.519	0.78	0.94	0.79	1.070	62.39	63.16	0.95
V122409A	0.626	0.87	1.07	0.71	1.101	56.55	55.16	1.05
V122409C	0.519	0.79	0.95	0.79	1.026	59.74	57.82	1.03
V181209A	0.575	0.83	1.01	0.75	1.061	57.65	57.82	1.00
V181209C	0.719	1.37	1.54	0.42	1.880	79.95	80.95	0.99
V181809A	0.611	1.24	1.38	0.52	1.766	87.08	88.78	0.99
V181809C	0.624	1.27	1.41	0.50	1.626	83.94	82.29	0.99
V241209A	0.582	1.20	1.33	0.55	1.584	77.56	77.62	1.03
V241209C	0.606	1.67	1.77	0.32	1.737	71.90	70.77	1.04

**Table A12:** Analysis results of test specimens reported by Johnson and Cafolla [26].

Specimen ID	*λ_L_*	*λ_G_*	*λ_I,1_*	*ρ _e_*	*ζ*	*V* (kN)	*V_T_* (kN)	*V/V_T_*
CW1	0.813	0.34	0.88	0.83	0.660	133.74	126.59	1.07
CW2	0.747	0.33	0.82	0.87	0.669	152.26	150.56	1.00
CW3	0.999	0.35	1.06	0.72	0.667	110.05	100.22	1.12

**Table A13:** Analysis results of test specimens reported by Peil [27].

Specimen ID	*λ_L_*	*λ_G_*	*λ_I,1_*	*ρ _e_*	*ζ*	*V* (kN)	*V_T_* (kN)	*V/V_T_*
SP1	0.996	0.26	1.03	0.74	1.077	224.99	225.00	1.00
SP2	1.136	0.35	1.19	0.64	1.202	213.74	215.30	0.98
SP3	1.222	0.44	1.30	0.57	1.324	205.69	209.50	0.97
SP4	0.783	0.30	0.84	0.85	0.999	232.31	230.80	1.02
SP5	0.897	0.41	0.99	0.76	1.095	222.07	220.50	1.02
SP6	0.981	0.53	1.11	0.68	1.177	218.68	220.00	0.99
SP2-2-400 1	1.066	0.16	1.08	0.71	0.981	84.29	80.25	1.05
SP2-2-400 2	1.066	0.16	1.08	0.71	0.981	84.29	88.13	0.95
SP2-2-800 1	1.084	0.33	1.13	0.67	1.060	178.87	178.88	1.00
SP2-2-800 2	1.084	0.33	1.13	0.67	1.060	178.87	177.75	1.01
SP2-3-600 1	0.751	0.24	0.79	0.89	1.121	302.98	301.50	1.00
SP2-3-600 2	0.751	0.24	0.79	0.89	1.121	302.98	308.63	0.98
SP2-3-1200 1	0.751	0.47	0.89	0.82	1.221	614.51	611.25	1.01
SP2-3-1200 2	0.751	0.47	0.89	0.82	1.221	614.51	625.13	0.98
SP2-4-800 1	0.593	0.31	0.67	0.96	1.056	603.28	601.50	1.01
SP2-4-800 2	0.593	0.31	0.67	0.96	1.056	603.28	603.38	1.01
SP2-4-1600 1	0.595	0.62	0.86	0.84	1.197	1218.89	1215.38	1.00
SP2-4-1600 2	0.595	0.62	0.86	0.84	1.197	1218.89	1227.00	0.99
SP2-8-800 1	0.270	0.24	0.36	1.00	1.330	1332.58	1308.38	1.01
SP2-8-800 2	0.270	0.24	0.36	1.00	1.330	1332.58	1374.75	0.97

**Table A14:** Analysis results of test specimens reported by Driver et al. [5].

Specimen ID	*λ_L_*	*λ_G_*	*λ_I,1_*	*ρ _e_*	*ζ*	*V* (kN)	*V_T_* (kN)	*V/V_T_*
G7A	0.834	0.37	0.91	0.81	1.084	2190.70	2299.82	0.92
G8A	0.834	0.37	0.91	0.81	1.084	2190.70	2155.05	0.98

**Table A15:** Analysis results of test specimens reported by Lee et al. [28].

Specimen ID	*λ_L_*	*λ_G_*	*λ_I,1_*	*ρ _e_*	*ζ*	*V* (kN)	*V_T_* (kN)	*V/V_T_*
L1	1.146	0.22	1.17	0.65	1.097	743.50	745.62	1.00
L2	1.401	0.23	1.42	0.50	1.209	624.38	625.56	1.00
L3	1.146	0.63	1.31	0.57	0.921	540.06	532.02	1.02
L4	1.401	0.56	1.51	0.44	1.052	480.55	474.73	1.01
I1	0.815	0.86	1.18	0.64	1.463	1302.50	1313.50	0.99
I2	1.126	1.26	1.69	0.35	1.475	565.15	565.66	1.00
G1	0.509	0.91	1.04	0.73	1.077	1087.70	1095.96	0.99
G2	0.515	1.14	1.25	0.60	1.392	916.69	912.94	1.01
G3	0.515	1.39	1.48	0.46	1.759	888.05	929.62	0.94

**Table A16:** Analysis results of test specimens reported by Moon et al. [7].

Specimen ID	*λ_L_*	*λ_G_*	*λ_I,1_*	*ρ _e_*	*ζ*	*V* (kN)	*V_T_* (kN)	*V/V_T_*
PG2	0.831	0.85	1.19	0.64	0.998	869.56	873.60	0.99
PG1	0.732	0.87	1.13	0.67	0.946	864.75	843.20	1.03
PG3	0.732	0.73	1.03	0.74	1.053	1052.21	1052.80	1.01

**Table A17:** Analysis results of test specimens reported by Moussa [29].

Specimen ID	*λ_L_*	*λ_G_*	*λ_I,1_*	*ρ _e_*	*ζ*	*V* (kN)	*V_T_* (kN)	*V/V_T_*
TP20-300-30	0.395	0.38	0.55	1.00	0.833	84.89	84.72	1.00
A20-410-30-N	0.263	0.45	0.52	1.00	0.899	122.74	125.09	0.99
A20-410-45-N	0.263	0.34	0.43	1.00	0.857	118.28	116.46	1.01
A20-505-30-N	0.263	0.56	0.62	0.99	0.915	153.69	154.23	0.99
A20-505-45-N	0.263	0.42	0.50	1.00	0.895	152.72	152.49	0.99
B20-305-30	0.403	0.51	0.65	0.97	1.006	233.51	233.20	1.00
B20-305-45	0.403	0.39	0.56	1.00	1.044	248.99	251.72	0.99
B20-505-45	0.403	0.64	0.76	0.90	0.976	335.82	329.34	1.06
B20-505-45-N	0.263	0.42	0.50	1.00	0.852	150.59	150.43	0.96

**Table A18:** Analysis results of test specimens reported by Wang et al. [30], Sause [8] and Hannebauer [31].

Scheme 1.	*λ_L_*	*λ_G_*	*λ_I,1_*	*ρ _e_*	*ζ*	*V* (kN)	*V_T_* (kN)	*V/V_T_*
W1	0.567	0.66	0.87	0.83	0.858	587.62	567.00	1.05
SC1	0.798	0.36	0.88	0.83	1.088	2127.47	2007.53	1.14
V1b	0.314	0.24	0.39	1.00	1.028	200.94	206.50	0.97
